# Thresholds of adversity for endocrine disrupting substances: a conceptual case study

**DOI:** 10.1007/s00204-024-03748-9

**Published:** 2024-05-05

**Authors:** Judy Choi, Stefanie Rotter, Vera Ritz, Carsten Kneuer, Philip Marx-Stoelting, Marize de Lourdes Marzo Solano, Angelika Oertel, Susanne Rudzok, Andrea Ziková-Kloas, Tewes Tralau, Andreas Hensel

**Affiliations:** German Federal Institute for Risk Assessment (BfR), Max-Dohrn-Straße 8-10, 10589 Berlin, Germany

**Keywords:** Endocrine disrupting chemicals, Human health, Risk assessment, Threshold, Pesticides, Adversity

## Abstract

For endocrine disrupting chemicals (EDC) the existence of “safe exposure levels”, that is exposure levels that do not present an appreciable risk to human health is most controversially discussed, as is the existence of health-based reference values. Concerns have been especially raised that EDCs might not possess a threshold level such that no exposure level to EDCs can be considered safe. To explore whether or not threshold levels can be identified, we performed a screening exercise on 14 pesticidal and biocidal active substances previously identified as EDCs in the European Union. The respective substances are ideal subjects for case studies to review for endocrine activity and disruptive potential following well-defined regulatory assessment based on solid data to effectually establish adversity as consequence of endocrine disruption. Dimethomorph, metiram and propiconazole for which the weight of evidence demonstrating endocrine disruption was the strongest were used as subjects for further study. Epoxiconazole was additionally selected as its effects on the endocrine system are extensive. For all four substances, analysis of the toxicological data clearly indicated thresholds of adversity below which no adverse effects mediated through an endocrine mechanism were observed. Particular emphasis was placed on mechanistic considerations including homeostasis and the concept of adversity. As a proof of concept this study provides evidence that like other substances of toxicological concern EDCs have threshold levels for adversity. While for some EDCs the respective thresholds might indeed be very low this shows that, data allowing, for other EDCs sufficiently protective reference values can be derived.

## Introduction

One of the major controversies associated with the risk assessment of endocrine disrupting chemicals (EDC) is whether or not there is a safe exposure level and if toxicological reference values like an acceptable daily intake (ADI) for such chemicals can be derived. It is frequently argued that “*there is currently no scientific evidence that can be relied upon to set a threshold value for EDCs with sufficient protective certainty.*” [e.g., (Chemtrust 2014)].

Notably, while repeatedly corroborated this claim does not result from the review of any current data but rather roots in the methodological limitations and data scarcity for EDCs at the time it was postulated. Scientifically any claim of “no threshold” would be rather daring actually as it severely challenges the bidirectional nature of chemical reactions that underlie toxicological effects and the thermodynamics they are based upon. This is not to say that thresholds can be too low as to be experimentally accessible or that in the absence of data the “no threshold”-postulate might be the more practical choice in order to proceed. However, this should not be mistaken as evidence when challenging scientific fundamentals such as the defining chemistry of dose–response relationships. Such claims should indeed always be based on solid data.

The interpretation of a “threshold” for EDCs can indeed be twofold. According to WHO/IPCS (2002), “*An endocrine disruptor is an exogenous substance or mixture that alters function(s) of the endocrine system and consequently causes adverse health effects in an intact organism, or its progeny, or (sub)populations*.” This definition was reinstated 10 years later in a re-assessment of the state of the science prepared for the United Nations Environment Programme and World Health Organization (WHO/UNEP 2012). It has also been adopted as the scientific basis for the definition of EDCs in the European Union (EU) in the context of its chemicals’ legislations. The three core elements from the definition of an EDC described above are the following: the presence of (i) the endocrine activity of a substance; (ii) an adverse effect caused by the substance in an intact organism or its offspring or future generations; and (iii) a biologically plausible link between the endocrine activity and the adverse effect. Taking these core elements into account, a threshold for endocrine disruption could be interpreted either as an exposure level, below which there is either no endocrine activity, or as the level of endocrine activity that can no longer be plausibly linked to an adverse effect or adversity. Biologically and toxicologically the latter interpretation clearly makes more sense as the first omits large parts of the underlying biochemistry and the capability of endocrine of circuits to self-regulate. However, this dual interpretation has led to many, in parts misleading, debates about thresholds for EDCs among experts but also in the public domain.

In an effort for clarification the European Commission’s Joint Research Centre (JRC) published a report “*Thresholds for Endocrine Disrupters and related Uncertainties*”, which states that thresholds of adversity for EDCs are likely to exist but may be very low (JRC 2013). Moreover, EFSA stated in its Scientific Opinion on the hazard assessment of EDs, that risk management decisions are ideally based on the consideration of available hazard and exposure data in the risk assessment and concluded that “*EDs can therefore be treated like most other substances of concern for human health and the environment, i.e., be subject to risk assessment and not only to hazard assessment*” (EFSA 2013). The discussion was refueled when (Brescia 2020), concluded “*that there is nothing special or unique about endocrine disruption or greater uncertainties in its assessment compared to other non-genotoxic forms of toxicity to justify adopting a non-threshold approach by default*” and, in consequence, proposed a risk-based approach for EDCs. At the time the proposal immediately entailed critical voices mainly on the grounds of the untenability of thresholds because of (i) the choice of endpoint being critical due to the huge variation of hormone efficacy on different endpoints and (ii) the differences in developing and adult organisms (Demeneix et al. 2020).

The ensuing discussion foremost strengthened the previously made observation though that the threshold debate mostly appears to be a methodological question. At its core lies the question if the respective data are sensitive and good enough to establish a “safe” (*i.e.*, non-adverse) threshold with sufficient certainty (Solecki et al. 2017; Zoeller et al. 2014). This is complicated further by the fact that it can be difficult to distinguish between apparent and true dose-thresholds for EDCs through empirical dose–response studies alone. Usually, additional information on the mechanistic aspects is required (Solecki et al. 2017).

However, a meaningful understanding of the underlying discussion not only requires (i) a common scientific understanding on endocrine biochemistry and toxicology but also (and in this context equally important); (ii) a grasp of the available information and methodologies for regulatory decision-making.

The main goal and purpose of any substance-related regulatory framework is to allow and warrant the safe use of substances as not to endanger human or environmental health. In line with this, various legislations lay down data requirements depending on the respective context of use. Usually, the amount of data required directly correlates with the anticipated exposure and bioactivity. In the EU it are hence and unsurprisingly active substances, that is those used for plant protection or with biocidal purposes, that require the most comprehensive set of data and which are also most thoroughly assessed and evaluated for potential hazards including endocrine disrupting properties. The data requirements for these active substances generally entail a large number of toxicity studies to be conducted in several mammalian species (Niemann et al. 2023). With such an extensive overall dataset, active substances provide a particularly good source of information for exploring data- and methodology-related challenges in toxicology. This also includes the question of potential existence of a threshold level for EDCs and their prospective regulatory use.

The endocrine disruption (ED) assessment of active substances evaluated under the Plant Protection Products Regulation [PPPR; Regulation (EC) No 1107/2009; EC 2009] and the Biocidal Products Regulation [BPR; Regulation (EU) No 528/2012; EU 2012] is mandatory and follows the criteria and principles set out in Regulation (EU) No 2018/605 (EU 2018b). To assist applicants and risk assessors in drafting and reviewing the ED assessment, respectively, ECHA and EFSA with collaboration of JRC published the “*Guidance for the identification of endocrine disruptors in the context of Regulations (EU) No 528/2012 and (EC) No 1107/2009*” (referred to from this point on as ECHA/EFSA ED guidance), which provides a tiered assessment approach and includes a template and an Excel file for the harmonized presentation of the assessment (ECHA/EFSA 2018). The ECHA/EFSA ED guidance lays out the data requirements (*i.e.*, experimental studies) for investigating adverse effects and endocrine activity. For appropriate toxicity studies it mainly refers to the Organisation for Economic Co-operation and Development (OECD) framework and testing strategy for endocrine disruptors (OECD 2018). Table 1 provides an overview of the existing toxicity studies or test guidelines considered relevant for human health ED assessment. For many pesticidal active substances reviewed in the EU, a number of the higher tiered studies (*i.e.*, Levels 4 and 5 in vivo studies under the OECD framework) were already conducted and are available for review. Levels 2 and 3 assays are often conducted if there are observations of endocrine effects from the higher tiered in vivo studies.

**Table 1:** Overview of the types of mammalian toxicity studies considered for the endocrine disruption assessment (under human health) of active substances evaluated under PPPR and BPR (table extracted from ECHA/EFSA 2018 with modifications)

	Test/information	Pathway addressed (with focus on E, A, T, and S-mediated modalities)	Most common data of pesticidal active substances available for review
Level 1 Existing data and existing or new non-test information	Physical and chemical properties of the active substance	–	✔
	All available toxicological data from (non-)standardized tests	–	✔
	Read-across, chemical categories, QSARs and other in silico predictions, and ADME model predictions	–	
Level 2 In vitro assays providing data about selected endocrine mechanism(s)/pathways(s)	Estrogen (OECD TG 493) or androgen receptor binding affinity (US EPA TG OPPTS 890.1150)	E	
	Estrogen receptor transactivation (OECD TG 455, ISO 19040–3), yeast estrogen screen (ISO 19040–1 & 2)	E	
	Androgen receptor transactivation (OECD TG 458)	A	
	Steroidogenesis in vitro (OECD TG 456)^a^	S	
	Aromatase assay (US EPA TG OPPTS 890.1200)^a^	S	
	Thyroid disruption assays (e. g., thyroperoxidase inhibition, transthyretin binding)	T	
	Retinoid receptor transactivation assays	–	
	Other hormone receptors assays as appropriate	–	
	High-throughput screens	–	
Level 3 In vivo assays providing data about selected endocrine mechanism(s)/pathway(s)	Uterotrophic assay (OECD TG 440)^b^	EA	
	Hershberger assay (OECD TG 441)^c^	EAS	
Level 4 In vivo assays providing data on adverse effects on endocrine relevant endpoints	Repeated dose 28-day study (OECD TG 407)^d^	EATS	✔
	Repeated dose 90-day study (OECD TG 408)^d^	EATS	✔
	Pubertal development and thyroid function assay in peripubertal male rats (US EPA TG OPPTS 890.1500)	EATS	
	Pubertal development and thyroid function assay in peripubertal female rats (US EPA TG OPPTS 890.1450)	EATS	
	Prenatal developmental toxicity study (OECD TG 414)	EATS	✔
	Combined chronic toxicity and carcinogenicity studies (OECD TG 451–3)^d^	EATS	✔
	Reproduction/developmental toxicity screening test (OECD TG 421)	EATS	
	Combined repeated dose toxicity study with the reproduction/developmental toxicity screening test (OECD TG 422)	EATS	
	Developmental neurotoxicity study (OECD TG 426)	EAS	
	Repeated dose dermal toxicity: 21/28-day Study (OECD TG 410)	EAS	
	Subchronic dermal toxicity: 90-day study (OECD TG 411)	EATS	
	28-Day (subacute) inhalation toxicity study (OECD TG 412)	EATS	
	Subchronic inhalation toxicity: 90-day study (OECD TG 413)	EATS	
	Repeated dose 90-day oral toxicity study in non-rodents (OECD TG 409)^d^	EATS	✔
Level 5 In vivo assays providing more comprehensive data on adverse effects on endocrine relevant endpoints over more extensive parts of the life cycle of the organism	Extended one-generation reproductive toxicity study (OECD TG 443)^d,e^	EATS	
	Two-generation reproduction toxicity study (OECD TG 416; using the most recent version from 2001)^d,e^	EATS	✔*

*ADME* absorption, distribution, metabolism and excretion; *BPR* biocidal products regulation, EATS *E* estrogen; *A* androgen; *T* thyroid; *S* steroidogenic; *PPPR* Plant Protection Products Regulation; *QSAR* quantitative structure–activity relationship

^a^According to ECHA/EFSA (2018), the results of the in vitro assays for the S-mediated modality along with the results of the E- and A-mediated modalities should be considered together in order to sufficiently conclude on the absence of S-mediated endocrine activity

^b^According to ECHA/EFSA (2018), the evaluation of E-mediated activity can be considered sufficiently investigated for the ED assessment if this study was conducted and results included in the dossier for review. Besides this study, the output data from the ToxCast ER Bioactivity Model (https://comptox.epa.gov/dashboard) would also be considered as sufficient evidence for the evaluation of E-mediated activity

^c^According to ECHA/EFSA (2018), the evaluation of A-mediated activity can be considered sufficiently investigated for the ED assessment if this study was conducted and results included in the dossier for review

^d^According to the ECHA/EFSA (2018), the evaluation of T-mediated adversity can be considered sufficiently investigated for the ED assessment if the T-mediated parameters covered in these studies have been measured and results included in the dossier for review

^e^According to the ECHA/EFSA (2018), the evaluation of EAS-mediated adversity can be considered sufficiently investigated for the ED assessment if all of the EAS-mediated parameters covered in either one of these studies have been measured and results included in the dossier for review

^*^It should be pointed out that while OECD TG 416 is a commonly conducted test for examining reproductive toxicity potential of a pesticidal active substance, there are incidences for a number of active substances, for which the OECD TG 416 was conducted prior to the version update in 2001. In such cases, the EAS-mediated parameters for adversity from the older OECD TG 416 would not be sufficiently examined for the ED assessment

Note: It is worth mentioning that as of 2023, there are no validated mechanistic test guidelines available for evaluating T-mediated activity

When there are indications of adverse effects and endocrine activity for an active substance in the available toxicity studies, the ECHA/EFSA ED guidance (ECHA/EFSA 2018) instructs to perform a mode-of-action (MoA) analysis to establish a biologically plausible link between endocrine activity and adversity. This analysis is often supported with the knowledge of existing Adverse Outcome Pathways (AOP) (Ankley et al. 2010). An active substance is identified as an EDC, if a biologically plausible link could be established between endocrine activity and adversity. Under PPPR and BPR, active substances that have been identified as EDCs hence comply with all three core elements of an EDC, *i.e.,* endocrine activity, adversity in intact organisms and/or offspring, and a biologically plausible link between endocrine activity and adversity.

The aim of this article was to analyze the available data and ED assessments for the already identified EDCs within the framework of the PPPR and the BPR in order to determine whether a threshold for endocrine adversity can be identified. After an initial screening exercise four active substances, that is dimethomorph, metiram, propiconazole and epoxiconazole, were selected and subjected to three detailed case studies. As such the study not only defines the prerequisites for deriving endocrine threshold levels, but also highlights the constraints under which determining a threshold level is not feasible.

## Materials and methods

### Retrieval of pesticidal active substances identified as endocrine disrupting chemicals (EDCs)

The list of identified EDCs under the PPPR and the BPR was retrieved directly from the websites of EFSA and ECHA, the responsible agencies for the evaluation and approval of active substances to be used in plant protection products and biocidal products, respectively.

EFSA has released an overview of the ED assessment of active substances evaluated under PPPR that is publicly available and regularly updated at https://www.efsa.europa.eu/sites/default/files/2021-06/overview-of-the-endocrine-disrupting-ed-assessment-of-pesticide-active-substances-as-in-line-with-the-criteria-introduced-by-commission-regulation-2018605.xlsx, accessible also via the associated website of EFSA: https://www.efsa.europa.eu/en/topics/topic/endocrine-active-substances. At the time of the analysis (conducted in summer of 2023), EFSA identified 9 substances as EDCs, 28 substances as non-EDCs; for 28 substances, waiving of the ED assessment was applied and for 31 substances, additional data are requested before a conclusion on the ED assessment could be made. For the purpose of this work, the 9 substances identified as EDCs were selected for the screening exercise.

Similarly, ECHA has published its endocrine disruptor assessment list that includes the substances under the REACH Regulation or the BPR that were or have been brought for discussion to ECHA’s Endocrine Disruptors Expert Group (EDEG; https://echa.europa.eu/ed-assessment). However, only biocidal active substances identified as EDs were marked in the list as “concluded”, while those who were considered to be non-ED as well as those that still are under discussion are marked as “under development”. This might be due to the fact that the Biocidal Product Committee—and not the EDEG—is responsible for recommendations on approval or non-approval of active substances to the European Commission (EC). By August 2023 three out of the 28 active substances evaluated by the EDEG under the BPR were identified as EDCs for human health (and hence subject to non-approval decisions by the European Commission). The compiled list of identified EDCs under PPPR and BPR (12 substances altogether) can be found in Table 2.

**Table 2:** List of identified endocrine disruptors under human health by EFSA (under PPPR) or ECHA (under BPR)

Chemical name	CAS no	Status of review	Selected for screening	Data sources
EFSA				
Asulam	3337–71-1	Final	Yes	EFSA Conclusion (EFSA 2021d): RAR and ED assessment: https://www.efsa.europa.eu/en/consultations/call/public-consultation-active-substance-asulam-regards-assessments
Benthiavalicarb isopropyl	413615–35-7	Final	Yes	EFSA Conclusion (EFSA 2021b): https://efsa.onlinelibrary.wiley.com/doi/epdf/10.2903/j.efsa.2021.6833
Clofentezine	74115–24-5	Final	Yes	EFSA Conclusion (EFSA 2021c): RAR: https://www.efsa.europa.eu/sites/default/files/consultation/consultation/Clofentezine_revised_RAR_EDNegl_exp_August_2020_public.zip
Dimethomorph	110488–70-5	Ongoing	Yes	EFSA Conclusion (EFSA 2023a): RAR and ED Assessment: https://connect.efsa.europa.eu/RM/s/publicconsultation2/a0l7U0000011ZX5/pc0175
Mancozeb	8018–01-7	Final	Yes	EFSA Conclusion (EFSA 2020): https://efsa.onlinelibrary.wiley.com/doi/epdf/10.2903/j.efsa.2020.5755
Metiram	9006–42-2	Final	Yes	EFSA Conclusion (EC 2023; EFSA 2023b): RAR and ED assessment: https://open.efsa.europa.eu/questions/EFSA-Q-2015-00589
Metribuzin	21087–64-9	Ongoing	Yes	EFSA Conclusion (EFSA 2023c): https://efsa.onlinelibrary.wiley.com/doi/10.2903/j.efsa.2023.8140
Thiabendazole	148–79-8	Final	Yes	EFSA Conclusion (EFSA 2022b): https://efsa.onlinelibrary.wiley.com/doi/epdf/10.2903/j.efsa.2022.7212
Triflusulfuron-methyl	126535–15-7	Final	Yes	EFSA Conclusion (EFSA 2022c): https://efsa.onlinelibrary.wiley.com/doi/epdf/10.2903/j.efsa.2022.7303
**ECHA**				
1-[[2-(2,4-dichlorophenyl)-4-propyl-1,3-dioxolan-2-yl]methyl]-1H-1,2,4-triazole (Propiconazole)	60207–90-1	Final	Yes	Opinion of the Biocidal Products Committee on the application for renewal of the approval of the active substance propiconazole for product type 8 (ECHA 2022): https://echa.europa.eu/documents/10162/2b615a3d-38d2-0087-31b6-dda6cfea6902
2,2-dibromo-2-cyanoacetamide (DBNPA)	10222–01-2	Final	Yes	Opinion of the Biocidal Products Committee on the application for approval of the active substance 2,2-Dibromo-2-cyanoacetamide (DBNPA) for product type 4 (ECHA 2021a): https://echa.europa.eu/documents/10162/085a4896-b067-bdbc-e38c-8f794e60e4f3
Cyanamide	420–04-2	Final	Yes	Opinion of the Biocidal Products Committee on the application for approval of the active substance Cyanamide for product type 3 (ECHA 2021b): https://echa.europa.eu/documents/10162/f5e04e73-afe6-4595-abda-864931b167bb and product type 18 (ECHA 2021c): https://echa.europa.eu/documents/10162/0c97e426-a0a0-4030-a2ec-abdd80ef1396

*BPR* biocidal products regulation; *ED* endocrine disruption; *PPPR* plant protection products regulation; *RAR* renewal assessment report

### Screening of the dataset used for the identification of active substances as EDCs

A systematic screening exercise was performed for each identified ED in order to determine the reliability of the data used as evidence for the identification of the substance as ED as well as the degree of uncertainty in the ED assessment itself. The following substances were evaluated: asulam-sodium (EFSA 2021d), benthiavalicarb (EFSA 2021b), clofentezine (EFSA 2021c), cyanamide (ECHA 2021b, 2021c), DBNPA (ECHA 2021a), dimethomorph (EFSA 2023a), mancozeb (EFSA 2020), metiram (EFSA 2023b), metribuzin (EFSA 2023c), propiconazole (ECHA 2022), thiabendazole (EFSA 2022b) and triflusulfuron-methyl (EFSA 2022c).

The data sources (namely assessment reports) used for the screening are presented in Table 2 and a template with a predefined set of criteria was created for the screening. The criteria were established based on the required evidence for the identification of an ED in accordance with the ECHA-EFSA (2018) guidance document with additional emphasis on the question whether the endocrine-mediated adverse effects exhibited a dose–response relationship or a threshold level (see Table 3).

**Table 3:** Criteria established for the screening exercise to determine reliability of the ED assessment and identify threshold levels of endocrine adversity

Criterion	Description
Sufficient investigation of endocrine activity(ies) with positive findings	Multiple assays/tests (in silico, in vitro or in vivo) were conducted to investigate endocrine activity, and more than one assay/test demonstrated positive endocrine activity (*e.g.,* with statistical significance)
Sufficient investigation of endocrine adversity(ies) with positive findings	Multiple toxicity studies were available for the assessment of endocrine adversity, and endocrine adversities were observed in toxicity study or studies (*e.g.,* with statistical significance)
Dose–response relationship of effect(s)	Positive trend for effect(s) seen with increasing doses
Effect(s) observed in more than one study	This demonstrates that the observed effects are most likely not due to chance finding
Effect(s) observed in multiple species	This demonstrates that the observed effects are not species-specific and therefore most likely to be relevant for human health
The biological plausibility between endocrine activity/activities and adversity is well-established	Mode of action analysis clearly shows a link between endocrine activity and adversity
Threshold level for the endocrine adversity could be determined	A NOAEL and LOAEL could be established for the observed endocrine effect(s)

*NOAE*L no observed adverse effect level, *LOAEL* lowest observed adverse effect level

The screening was conducted by at least two toxicology experts, and the outcomes of the screening were compared. Any conflict or disagreement in the screening was resolved either by internal discussion or involvement of a third toxicology expert.

### Substance selection and presentation for case studies

From the outcomes of the screening exercise, three substances (dimethomorph, metiram and propiconazole) were selected as case studies as they had the most suitable data for the ED assessment (rf. to Sect. 3.1). Epoxiconazole was additionally included in the case study of propiconazole as its effects on the endocrine system are extensive, however a formal ED assessment according to the ECHA/EFSA ED guidance (ECHA/EFSA 2018) was never performed. They were combined in one case study as they are both triazoles with comparable modes of action allowing a grouping approach.

The selected substances cover different specific endocrine modalities: dimetomorph is an anti-androgen (A), metiram causes thyroid-mediated (T) adverse effects and propiconazole and epoxiconazole have multiple putative endocrine modes of action (including certainly S).

Each case study entails a background of the substance, the evaluation process under EFSA or ECHA, conclusion drawn on the ED assessment and a detailed review of all the toxicity studies, in which endocrine-mediated adverse effects relevant for the identification of the active substance as ED were observed. Particular focus was given on whether a dose–response relationship was observed and if a no-/lowest-observed-adverse-effect-level (NOAEL/LOAEL) could be established for each of the relevant adverse effects.

## Results

### Outcome of the screening exercise

An overview of the screening exercise is shown in Table 4. Following the ECHA/EFSA ED guidance (ECHA/EFSA 2018), sufficient investigation of endocrine adversity, which is the first criterion for identifying a substance as an ED according to WHO’s definition (WHO/IPCS 2002), was demonstrated in all of the 12 substances identified as ED and included in the screening exercise.

**Table 4:** Summary of the screening exercise of the identified EDs (bold indicates substance selected as a case study)

Criterion	2,2-Dibromo-2-cyanoacetamide	Asulam	Benthiavalicarb	Clofentezine	Cyanamide	Dimethomorph	Mancozeb	Metiram	Metribuzin	Propiconazole	Thiabendazole	Triflusulfuron-methyl
Sufficient investigation of endocrine activity(ies) with positive findings	p	p	N	Y	Y	**p**	N	**Y**	Y	**Y**	N	Y
Sufficient investigation of endocrine adversity(ies) with positive findings	p	Y	Y	Y	Y	**Y**	Y	**Y**	Y	**Y**	Y	Y
Dose–response relationship of endocrine adverse effect(s)	p	p	p	p	p	**Y**	Y	**Y**	Y	**Y**	p	Y
Effect(s) observed in more than one study	Y	Y	Y	Y	Y	**Y**	Y	**Y**	Y	**Y**	Y	Y
Effect(s) observed in multiple species	Y	Y	Y	N	Y	**Y**	Y	**Y**	Y	**Y**	Y	Y
The biological plausibility between endocrine activity/activities and adversity is well-established	Y	p	p	Y	Y	**Y**	Y	**Y**	p	**Y**	p	p
Threshold level for the endocrine adversity could be determined	Y	p	Y	p	Y	**Y**	Y	**Y**	Y	**Y**	Y	Y
Degree of uncertainties^a^	+	+ + +	+ + +	+ +	+ +	** + **	+	** + **	+ +	** + **	+ + +	+ +

*Y* yes; *N* no; p: partially (This applies when there are differences or uncertainties in the assessment, e.g., dose–response relationship observed for one effect but not for another effect.)

^a^ +  +  + : high; +  + : moderate; + : low

However, sufficient investigation of endocrine activity, the second criterion of an ED according to the WHO, was only achieved for a few substances. This is mainly due to lack of validated methods for determining thyroid activity or because the respective substances were approved prior to standardized ED-testing and assessment. Nevertheless, for all 12 substances, there is an indication of potential endocrine activity.

Notably, biological plausibility of the link between endocrine activity and adversity, the third and last criterion of an ED according to the WHO, could likewise be established only in some of the substances. For the other substances the respective MoA analysis was not conclusive, albeit not to an extent that a biological link could have been excluded (see Table 4 for more details).

The targeted screening resulted in the identification of three substances for which there was sufficient data as to investigate if and what threshold level could be observed. Namely, this were dimethomorph, metiram and propiconazole.

### Case studies

#### Dimethomorph—an anti-androgen

Dimethomorph (CAS 110488–70-5) has been approved as an active substance for the use on potatoes in plant protection products (PPPs) within the European Union (EU) since 2007. It belongs to the morpholine fungicides with respective PPPs being authorized in all EU Member States. For the purpose of renewal of approval an initial draft Renewal Assessment Report (RAR) was prepared in 2017 by the Netherlands as the Rapporteur Member State (RMS) and Germany as Co-Rapporteur Member State (Co-RMS) (Netherlands 2017). Dimethomorph has been under the EU’s Pesticides Peer Review procedure since 2018 with an amendment on the assessment of the endocrine disrupting potential in accordance to the ECHA/EFSA ED guidance added to the RAR in 2019 (ECHA/EFSA 2018).

Under the harmonized classification and labeling (CLH) Dimethomorph is classified as Repro 1B (H360F) and as such meets the cut-off criteria for renewal of approval as set out in Regulation (EC) No 1107/2009. It further fulfills the endocrine disrupting criteria according to the “*Overview of the endocrine disrupting (ED) assessment of pesticide active substances in line with the criteria introduced by Commission Regulation 2018/605*” (EFSA 2022a).

Together this makes dimethomorph an ideal candidate for a case study as it is subject to a comprehensive dataset spanning multiple studies in two different species (dog and rat) in conjunction with a plausible endocrine mode of action for the observed adversity.

In line with the regulatory process the initial ED assessment for dimethomorph was performed by the RMS with the result subsequently being put forward for peer-review and discussion by the Pesticide Peer Review Expert Panel at EFSA.

The underlying dataset predominantly consists of standard OECD guideline studies together with supplementary data from the ToxCast program (EPA 2022). Briefly, dimethomorph was found to fulfill the criteria of an endocrine disruptor with sufficient experimental animal data to indicate anti-androgenic adversity from dimethomorph exposure. Observed effects include decreased prostate weight and concomitant histopathological findings (fibrosis and prostatitis), increased incidence of testicular effects (*e.g.*, interstitial cell proliferation, hyperplasia and adenoma) and altered sexual development (delayed preputial separation and decreased anogenital distance in males) in dogs or rats, respectively. The effects considered relevant for the identification of dimethomorph as ED are listed in Table 5.

**Table 5:** Reported findings on dimethomorph related to male reproduction/sexual development—effects considered relevant for ED identification are marked in bold

No	Effect	NOAEL	LOAEL	Unit(s)	Dose–response	Remarks	Study
**(1)**	**Prostate weight ↓ (absolute/relative)**	**450** **(15)**	**1350** **(43)**	**ppm** **(mg/kg bw/d)**	**Yes**	4/sex/group; no sig. change in bw among all groups	13-week dog study, OECD TG 409, Study ID 7 (Anonymous 1986)
**(2)**	**Prostate histopathology, ↑ fibrosis and prostatitis**	**450** **(15)**	**1350** **(43)**	**ppm** **(mg/kg bw/d)**	**Yes**	4/sex/group; 2 with prostatitis and 4 with fibrosis (vs. 0 in other groups for this effect)	13-week dog study, OECD TG 409, Study ID 7 (Anonymous 1986)
**(3)**	**Prostate weight ↓ (absolute/relative)**	**450** **(14.7)**	**1350** **(44.6)**	**ppm** **(mg/kg bw/d)**	**Yes (but not w/ low dose)**	4/sex/group; no sig. change in bw among all groups	52-week dog study, OECD TG 409, study ID 8 (Anonymous 1988)
**(4)**	**Prostate histopathology, change, interstitial fibrosis**	**450** **(14.7)**	**1350** **(44.6)**	**ppm** **(mg/kg bw/d)**	**Yes**	4/sex/group Increasing severity in effect (from minimal to moderate)	52-week dog study, OECD TG 409, study ID 8 (Anonymous 1988)
(5)	Testis weight ↑ (absolute/adjusted)	150 (4.9)	450 (14.7)	ppm	Yes	4/sex/group; no sig. change in bw among all groups; no histopathological correlation	52-week dog study, OECD TG 409, study ID 8 (Anonymous 1988)
**(6)**	**Testis histopathology ↑, interstitial cell proliferation (hyperplasia)**	**750** **(36.2)**	**2000** **(99.9)**	**ppm** **(mg/kg bw/d)**	**Yes**	20/sex/group; method of EU Testing Method B 30	2-year rat study, study ID 10, (Anonymous 1991c)
**(7)**	**Testis histopathology ↑, interstitial cell adenomas**	**750** **(36.2)**	**2000** **(99.9)**	**ppm** **(mg/kg bw/d)**	**Unclear due to limited sample size**	20/sex/group; method of EU Testing Method B 30 Incidence of 2/5/4/6 with 0/200/750/2000 ppm	2-year rat study, study ID 10, (Anonymous 1991c)
**(8)**	**Adult, testis histopathology change**	**750** **(33.9)**	**2000** **(94.6)**	**ppm** **(mg/kg bw/d)**	**Yes**	50/sex/group; Method of EU Testing Method B32 Equivocal effect on the incidence of testicular interstitial cell adenomas within HCD	2-year rat study, Carc. / OECD 451, study ID 11, (Anonymous 1991b)
**(9)**	**Offspring (F1), age at preputial separation ↑**	**800**	**1600**	**ppm**	**Yes (but not w/ low dose)**	“Effects seen at 800 ppm attributed to growth retardation. In the high dose, slight effect on age of preputial separation could be attributed to an anti-androgenic activity.”	EOGRTS, study ID 14 (Anonymous 2014c) (HCD: (Anonymous 2018))
**(10)**	**Offspring (F1), anogenital distance (AGD) ↓**	**800**	**1600**	**ppm**	**Yes**	Effect more observable in male than female pups	EOGRTS, study ID 14 (Anonymous 2014c) (HCD: (Anonymous 2018))
**(11)**	**Offspring (F1), Anogenital index (AGI) ↓**	**800**	**1600**	**ppm**	**Yes (males only)**	Adjusted for bw; effect observed in males only	EOGRTS, study ID 14 (Anonymous 2014) (HCD: (Anonymous 2018))
(12)	Adult (F1), epididymis weight, absolute ↓	800	1600	ppm	Yes	Cohort 1A: -9% compared to control; no difference in relative wt No histopath. correlates	EOGRTS, study ID 14 (Anonymous 2014c) (HCD: (Anonymous 2018))
(13)	Adult (F1), prostate weight, absolute, ↓	–	300	ppm	Yes	Cohort 1A: -11% compared to control (at LOAEL); not statistically. sig. in rel. wt No histopath. correlates	EOGRTS, study ID 14 (Anonymous 2014c) (HCD: (Anonymous 2018))
**(14)**	**Cohort 1B, prostate weight, absolute ↓**	**300** **–**	**800**	**ppm**	**Yes**	At 300 ppm, the ↓ was considered not adverse as no stat. sig. diff. in relative wt was observed and the ↓ was most likely due to ↓ bw Mid- and high doses (i.e., at 800 ppm and above) considered treatment-related	EOGRTS, study ID 14 (Anonymous 2014c) (HCD: (Anonymous 2018))
**(15)**	**Cohort 1B, prostate weight, relative ↓**	**300**	**800**	**ppm**	**Yes**	Treatment-related	EOGRTS, study ID 14 (Anonymous 2014c) (HCD: (Anonymous 2018))
(16)	Adult (F0), prostate weight, absolute ↓	800	1600	ppm	Yes	− 12% compared to control “*Prostate wt. was within the range of HCD (0.796—1.228 g) and there was no histopath. correlate. Also, the rel. prostate wt. did not show a sig. decrease. Therefore, the decrease of prostate wt. was related to the slightly but not sig. reduced terminal bw (−4%) in this test group*” (Netherlands 2017)	EOGRTS, study ID 14 (Anonymous 2014c) (HCD: (Anonymous 2018))
(17)	Adult (F0), seminal vesicles weight, absolute, ↓	300	800	ppm	Yes	“*These wt. were within the range of HCD (0.905—1.426 g) and there were no histopath. correlates. Also, the relative wts. of the seminal vesicle did not show a significant decrease.*” (Netherlands 2017)	EOGRTS, study ID 14 (Anonymous 2014c) (HCD: (Anonymous 2018))
**(18)**	**Adult (F1), seminal vesicles weight, absolute, ↓**	**300**	**800**	**ppm**	**Yes**	Cohort 1A Considered as treatment-related; no HCD of F1 for comparison but outside the HCD for F0 males	EOGRTS, study ID 14 (Anonymous 2014c) (HCD: (Anonymous 2018))
**(19)**	**Adult (F1), seminal vesicles weight, relative ↓**	**800**	**1600**	**ppm**	**Yes**	Cohort 1A Considered as treatment-related	EOGRTS, study ID 14 (Anonymous 2014c) (HCD: (Anonymous 2018))
**(20)**	**Cohort 1B, seminal vesicles weight, absolute ↓**	**300**	**800**	**ppm**	**Yes**	Considered as treatment-related	EOGRTS, study ID 14 (Anonymous 2014c) (HCD: (Anonymous 2018))
**(21)**	**Cohort 1B, seminal vesicles weight, relative ↓**	**300**	**800**	**ppm**	**Yes**	Considered as treatment-related	EOGRTS, study ID 14 (Anonymous 2014c) (HCD: (Anonymous 2018))
(22)	Adult (F1), testis weight, relative ↑	300	800	ppm	Yes	Cohort 1A No sig. diff on abs. wt No histopath. correlates	EOGRTS, study ID 14 (Anonymous 2014c) (HCD: (Anonymous 2018))
(23)	Cohort 1B, testis weight, relative ↑ i	300	800	ppm	Yes	Most likely not treatment-related as effect not observed in cohort 1A	EOGRTS, study ID 14 (Anonymous 2014c) (HCD: (Anonymous 2018))
(24)	Cohort 1B, testis weight, absolute ↑	800	1600	ppm	Yes	Most likely not treatment-related as effect not observed in cohort 1A	EOGRTS, study ID 14 (Anonymous 2014c) (HCD: (Anonymous 2018))
**(25)**	**Receptor binding, Androgen receptor ↑** **Anti-androgenic activity** **(YAS-assay)**	**1.00E-04** **(10**^**–5**^**)**	**1.00E-03** **(10**^**–4**^**)** **Effective conc**	**mol/L**	**Yes**	(Netherlands 2017): “*A reproducible inhibition of the androgen effect compared to 5× 10*^−9^* mol/L 5α-dihydrotestosterone (partly or total suppression of expected color development) was observed at a concentration of 10*^***−4***^* mol/L. The inhibition was above 20% and therefore dimethomorph is considered to antiandrogenic.***”** Only 2 replicate experiments, no guideline but GLP-compliant	AR Binding Assay, Saccharomyces cerevisiae (Woitkowiak 2011)

*AR* androgen receptor; *bw* body weight; *carc*. carcinogenicity; *EOGRTS* extended one-generation reproductive toxicity study; *GLP* good laboratory practice; *HCD* historical control data; *LOAEL* lowest observed (adverse) effect level; *NOAEL* no observed (adverse) effect level; sig. = significant; ↑ = increase; ↓ = decrease

In addition to the evidence in vivo, anti-androgenic activity of dimethomorph was also observed in the yeast androgen screening (YAS) assay (Table 5) and there was an indication of antagonistic activity in the ToxCast androgen receptor (AR) pathway model (Table 6). Together this was found to sufficiently warrant a biological plausible link between adversity and activity according to ECHA/EFSA’s ED guidance (ECHA/EFSA 2018), ED criteria for EAS-modalities were met (EFSA 2023a).

**Table 6:** Predictions from the ToxCast models for dimethomorph

Model	Receptor	Agonist	Antagonist	Binding
CERAPP Potency Level (consensus)	Estrogen	0	0	0
ToxCast Pathway Model (area under the curve)	Androgen	0	0.237*	–
CERAPP Potency Level (from literature)	Estrogen	Inactive	Inactive	Inactive
ToxCast Pathway Model (area under the curve)	Estrogen	0	0	–

^*^indicates the results relevant for the identification of dimethomorph as an endocrine disruptor. Results retrieved on November 23, 2023 (EPA 2022)

The corresponding MoA analysis presented a probable AOP where the molecular initiating event of antagonistic binding to the AR ultimately results in feminization or incomplete development of primary and accessory male organs as well as to impairment of reproductive capacity ((AOP-19,2021) (Manibusan and Touart 2017)). Substance triggered altered sexual development due to binding to the AR is also supported by other related AOPs (e.g., “*androgen receptor antagonism leading to short anogenital distance (AGD) in male (mammalian) offspring*”) (AOP-306,2021). Based on the weight of evidence dimethomorph has hence to be considered an endocrine disruptor as laid down in Regulation (EU) No 2018/605 (EU 2018b) and according to the ED guidance (ECHA/EFSA 2018).

**Fig. 1:**
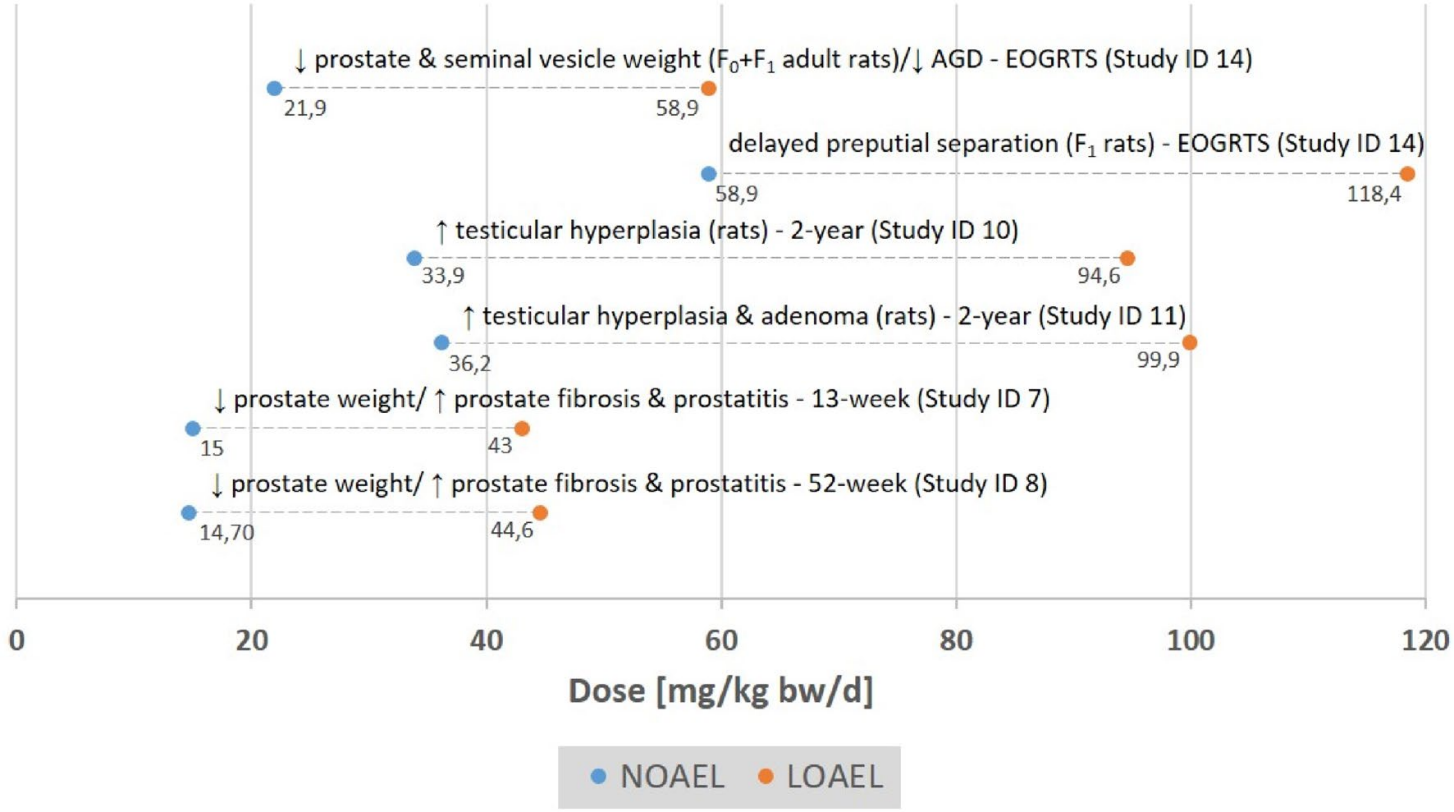
Overview of the NOAELs/LOAELs for the endocrine adverse effects of dimethomorph relevant for the identification

The studies for the ED assessment of dimethomorph are all compliant in accordance with the principles of Good Laboratory Practice (GLP) and with respective OECD Test Guidelines (see Table 5 for the guidelines used). Data cover multiple species and various exposure scenarios that comprise subchronic as well as chronic toxicity. For each study with reported endocrine adversity there were 3 doses along with a control group. The intervals between doses were appropriately spaced between 3- and fivefold. Therefore, the studies are considered reliable and suitable for a threshold assessment. Table 5 presents an overview of the findings. The no- or lowest-observed-adverse-effect-levels (NOAEL/LOAEL) for each of the relevant endocrine adverse effects can be seen in Fig. 1.

A clear dose–response relationship with statistical significance at the LOAEL could be observed for nearly all of the adverse effects (*e.g.*, organ weights, anogenital distance). For example, male dogs exposed to the LOAEL of 43 and 44.6 mg/kg bw/d for 13 and 52 weeks exhibited a significant decrease in absolute and relative prostate weight, whereas at the NOAEL of 14.7–15 mg/kg bw/d a decrease was allegedly noticeable but not statistically significant anymore. Likewise, the histopathological findings (*e.g.*, prostatitis and fibrosis) accompanying the decreased prostate weight at the LOAEL at 13 and 52 weeks were not observed at all or only to a much lesser degree at the NOAEL. In dog the respective histopathological findings were less clear. However, given the sample size of 4 animals per dose any in-depth interpretation beyond a qualitative analysis appears inadequate due to the very limited significance. Again, a clear dose–response relationship between exposure to dimethomorph and altered sexual development was observed with regard to altered sexual development in the extended one-generation reproductive toxicity study (EOGRTS; OECD TG 443) in rat. In male pups this comprised anogenital distance and day of preputial separation as well as decrease of weight of prostates and seminal vesicles. Also, there was an increasing trend between exposure to dimethomorph and testicular hyperplasia and/or adenoma.

For dimethomorph the overall evidence therefore strongly supports a threshold for dimethomorph-induced adversity via antagonism of androgen signaling.

### Propiconazole and epoxiconazole—multiple putative endocrine modes of action

Triazoles belong to the most frequently used groups of agricultural fungicides because of their efficacy as well as their curative and eradicative properties. The fungicidal activity of azole fungicides roots in their inhibition of the fungal cytochrome P-450-containing (CYP)-enzyme ergosterol synthetase.

Propiconazole (CAS 60207–90-1) is classified as Repro 1B due to its effects on developmental toxicity. Further it has been identified as endocrine disruptor based on an assessment according to the ECHA/EFSA ED guidance (ECHA/EFSA 2018). As active substance it hence fulfills the exclusion criteria of the PPPR and the BPR resulting in non-renewal and listing as candidate for substitution, respectively.

Similarly, epoxiconazole (CAS 106325–08-0) is classified as Repro 1B due to developmental toxicity. It is also classified as carcinogenic Carc. 2. Consequently, its approval as active substance for the use in PPPs has been suspended. Although a formal ED assessment according to the ECHA/EFSA ED guidance (ECHA/EFSA 2018) was never performed, the data on its effects on the endocrine system are so extensive that they were included into this study.

Notably the aforementioned classification of propiconazole and epoxiconazole as Repro 1B comes from the observed ability to cause malformations such as cleft palate and other cranio-facial defects. Biologically such defects are often caused by aberrant retinoic acid (RA) metabolism, for example increased concentrations due to inhibition of CYPs 26 or 3A4 (Robinson et al. 2012). In the case of epoxiconazole also further possible modes of action are discussed (ECHA 2012). Without elucidation of the mode(s) of action for these effects and classification of RA signaling as endocrine or non-endocrine still being subject to scientific debate an assessment of these effects following the ECHA/EFSA ED guidance was not attempted (ECHA/EFSA 2018).

However, apart from effects on RA metabolism the varying ability of azole fungicides to inhibit CYP enzymes can also lead to side effects on hormone production. Unsurprisingly, ED properties of azoles have hence been discussed for quite some time (Marx-Stoelting et al. 2014; Taxvig et al. 2013). The CYP-mediated effects (*i.e.,* via CYP19A1 inhibition) of azole fungicides potentially affect a range of endocrine pathways and comprise substance-induced decrease in estradiol or increase in testosterone with subsequent changes of levels of follicle stimulating hormone (FSH) or adrenocorticotropic hormone (ACTH), respectively. The resulting hormonal imbalances can affect fertility or induce tumor formation in endocrine organs (*e.g.*, ovaries and adrenals for epoxiconazole). Apparently female animals seem more sensitive, which is in line with predominant promiscuous interference with sex hormone metabolism and signaling.

Concomitantly effects reported for propiconazole are estrous cycle disturbance and altered testis weights (Tables 7, 8), however there is no affection of thyroid-related modalities. Epoxiconazole, likewise, shows a number of adverse effects on reproduction. In females these include, among others, ovarian cysts, ovarian theca–granulosa tumors, adrenal cortex adenoma, vaginal hemorrhages during pregnancy and increased placenta weight, while in males sex hormone levels for testosterone, ACTH and FSH can be critically affected (Tables 9 and 10). However, as before propiconazole, epoxiconazole does not show primary thyroid toxicity.

**Table 7:** Reported findings on propiconazole related to female reproduction - effects considered relevant for ED identification are marked in bold

No	Effect	NOAEL	LOAEL	Unit(s)	Dose–response	Remarks	Study
**(1)**	**Estrous cycle disturbance**	**500**	**2500**	**ppm**	**Yes**	Only in young immature animals (1–2 week post vaginal opening) 500 ppm: lowest average intake**~ **42 mg/kg bw/d 2500 ppm: lowest average intake ~ 192 mg/kg bw/d	2-generation study, rat (Anonymous 1985)
(2)	No effects	–	–	–	n.d	No binding to estrogen receptor	In vitro estrogen receptor binding assay (Anonymous 2012a)
**(3)**	**Aromatase inhibition**	**1**	**10**	**µM**	**Yes**		In vitro aromatase assay ASB2010-14396 (Sanderson et al. 2002)
**(4)**	**Aromatase inhibition**	**0,1**	**1**	**µM**	**Yes**		In vitro aromatase assay ASB2020-25714 (Ohno et al. 2004)
**(5)**	**Progesterone levels ↓**	**25**	**50**	**µM**	**Yes**		H295R steroidogenesis assay (Taxvig et al. 2013)
**(6)**	**Estradiol levels ↓**	**–**	**0.1**	**µM**	**Yes**	0.1 µM: lowest dose tested	H295R assay (Taxvig et al. 2013)
(7)	No effects	–	–	–	n.d	No estrogenic effects	Uterotrophic assay ASB2020-25717 (Anonymous 2012b)
**(8)**	**Aromatase inhibition (after 2 h exposure)**	**–**	**1**	**µM**	**Yes**	1 µM: lowest dose tested	In vitro aromatase assay (Laville et al. 2006)
**(9)**	**Aromatase inhibition**	**–**	**1**	**µM**	**Yes**	1 µM: lowest dose tested	In vitro aromatase assay, ASB2010-14400 (Vinggaard et al. 2000)

LOAEL = lowest observed (adverse) effect level; n.d. = not detected; NOAEL = no observed (adverse) effect level; ↓ = decreased

**Table 8:** Reported findings on propiconazole related to male reproduction - effects considered relevant for ED identification are marked in bold

No	Effect	NOAEL	LOAEL	Unit(s)	Dose–response	Remarks	Study
(1)	Pre-pubertal testosterone levels ↓	–	4	mg/kg bw/d	No (not observed at higher dose)	Only 2 dose levels; alterations did not cause changes in the function of the reproductive organs	2-gen rat, male rats only (Vieira et al. 2017)
(2)	Seminal vesicle weight	–	4	mg/kg bw/d	No (not observed at higher dose)	Only 2 dose levels;	Reprotox, male rats only (Costa et al. 2015)
(3)	Sexual behavior	4	20	mg/kg bw/d	Yes	Only 2 dose levels	Reprotox, male rats only (Costa et al. 2015)
**(4)**	**Relative testis weight↑**	**1200**	**6000**	**ppm**	**Yes**	1200 ppm: ~ 76 mg/kg bw/d 6000 ppm: ~ 462 mg/kg bw/d	90d oral, rat (Anonymous 1979)
**(5)**	**Absolute testis weight ↓**	**500**	**2500**	**ppm**	**Yes**	500 ppm: lowest average intake ~ 42 mg/kg bw/d 2500 ppm: lowest average intake ~ 192 mg/kg bw/d	OECD TG 416 (Anonymous 1985)
**(6)**	**Testosterone synthesis**	**10**	**30**	**µM**	**Yes**		OECD TG456, 48 h, Steroidogenesis assay (Goetz et al. 2009)

*NOAEL* no observed (adverse) effect level; *LOAEL* lowest observed (adverse) effect level; bw = body weight; ↑ = increase; ↓ = decrease

**Table 9:** Reported findings of epoxiconazole related to female reproduction - effects considered relevant for ED identification are marked in bold

No	Effect	NOAEL	LOAEL	Unit(s)	Dose–response	Remarks	Study
**(1)**	**Ovarian cysts**	**9**	**44**	**mg/kg bw/d**	**Yes**		Rat, chronic (24 ms) TOX2003-1844 (Anonymous 1992d)
**(2)**	**Ovarian cysts**	**2**	**8**	**mg/kg bw/d**	**Yes**		Rat, carc. (24 mos) TOX2003-1845 (Anonymous 1992g)
**(3)**	**Ovary: theca–granulosa tumors**	**9**	**44**	**mg/kg bw/d**	**Yes**		Rat, chronic (24 mos) TOX2003-1844 (Anonymous 1992d)
**(4)**	**Ovary: theca–granulosa tumors**	**8**	**45**	**mg/kg bw/d**	**Yes**		Rat, carc. (24 mos) TOX2003-1845 (Anonymous 1992g)
(5)	Senile involution of ovary	8 (9/50)	45 (3*/50)	mg/kg bw/d	Yes	Not adverse, inverse correlation	Rat, carc. (24 mos) TOX2003-1845 (Anonymous 1992g)
**(6)**	**Adrenal cortex adenoma**	**45**	**90**	**mg/kg bw/d**	**Yes**		Rat, carc. (24 mos) TOX2003-1845 (Anonymous 1992g)
**(7)**	**Vaginal hemorrhages during pregnancy**	**2.3**	**23**	**mg/kg bw/d**	**Yes**		Rat, 2gen TOX2003-1847 (Anonymous 1992f)
**(8)**	**Vaginal hemorrhages during pregnancy**	**60**	**75**	**mg/kg bw/d**	**Yes**		Rat, devtox, TOX2003-1852 (Anonymous 2001)
(9)	Blood in bedding during pregnancy	20 (1/20)	80 (1/20)	mg/kg bw/d	Yes		Rabbit, devtox, TOX2003-1851 (Anonymous 1990b)
**(10)**	**Gestation index ↓**	**2.3**	**23**	**mg/kg bw/d**	**Yes**		Rat, 2gen TOX2003-1847 (Anonymous 1992f)
**(11)**	**Pregnancy > 22 d**	**2.3**	**23**	**mg/kg bw/d**	**Yes**		Rat, 2gen TOX2003-1847 (Anonymous 1992f)
(12)	Placental weight ↑	**–**	20	mg/kg bw/d	Yes	Not adverse, indicative starting at lowest dose	Rat, devtox, TOX2003-1848 (Anonymous 1989b)
(13)	Placental weight ↑	5	15	mg/kg bw/d	Yes	Not adverse, indicative	Rat, devtox, TOX2003-1849 (Anonymous 1990c)
(14)	Placental weight ↑	400	1000	mg/kg bw/d	Yes	Not adverse, indicative	Rat dermal devtox, TOX2003-1850 (Anonymous 1993)
(15)	Placental weight ↓	–	5	mg/kg bw/d	(Yes)	Not adverse, indicative, not statistically sig	Rabbit, devtox TOX2003-1851 (Anonymous 1990b)
(16)	CYP ↑	**–**	~ 5	mg/kg bw/d	Yes		Rat, enzyme induction study, TOX2003-1860 (Anonymous 1987)
(17)	Microsomal protein ↑	~ 22.5	~ 100	mg/kg bw/d	Yes		Rat, enzyme induction study, TOX2003-1860 (Anonymous 1987)
(18)	CYP ↑	**–**	~ 1.5	mg/kg bw/d	Yes		Mouse, enzyme induction study, TOX2003-1859 (Anonymous 1990a)
(19)	Microsomal protein ↑	~ 4.5	~ 13.5	mg/kg bw/d	Yes		Mouse, enzyme induction study, TOX2003-1859 (Anonymous 1990a)
**(20)**	**Dehydroepiandrosterone ↑, in diestrus/proestrus**	**–**	**65**	**mg/kg bw/d**	**No**		Rat, hormone conc. study, TOX2003-1857 (Anonymous 1992a)
**(21)**	**Androstenedione ↑, in diestrus/proestrus**	**–**	**65**	**mg/kg bw/d**	**Yes**		Rat, hormone conc. study, TOX2003-1857 (Anonymous 1992a)
**(22)**	**Estradiol ↓, in diestrus/proestrus**	**–**	**65**	**mg/kg bw/d**	**Yes**		Rat, hormone conc. study, TOX2003-1857 (Anonymous 1992a)
**(23)**	**LH ↑, in diestrus**	**–**	**65**	**mg/kg bw/d**	**Yes**		Rat, hormone conc. study, TOX2003-1857 (Anonymous 1992a)
**(24)**	**Prolactin ↓, in proestrus**	**–**	**65**	**mg/kg bw/d**	**Yes**		Rat, hormone conc. study, TOX2003-1857 (Anonymous 1992a)
**(25)**	**ACTH ↑, in diestrus, 4 weeks**	**94**	**160**	**mg/kg bw/d**	**Yes**		Rat, hormone conc. study, TOX2003-1857 (Anonymous 1992a)
**(26)**	**Aldosterone ↓, in diestrus/proestrus**	**–**	**94**	**mg/kg bw/d**	**Yes**		Rat, hormone conc. study, TOX2003-1857 (Anonymous 1992a)

*ACTH* adrenocorticotropic hormone; *carc*. carcinogenicity; *LH* luteinizing hormone; *LOAEL* = lowest observed (adverse) effect level; mos = months; *NOAEL* no observed (adverse) effect level; sig- = significant; ↑ = increase; ↓ = decrease

**Table 10:** Reported findings of epoxiconazole related to male reproduction - effects considered relevant for ED identification are marked in bold

No	Effect	NOAEL	LOAEL	Unit(s)	Dose–response	Remarks	Study
(1)	Amyloid deposition in testes	35	72	mg/kg bw/d	Yes		Mouse chronic/carc TOX2003-1846 (Anonymous 1992f)
**(2)**	**Reduced male fertility index**	**2.3**	**23**	**mg/kg bw/d**	**Yes**		Rat, 2gen TOX2003-1847 (Anonymous 1992c)
**(3)**	**Prolonged cohabitation time**	**2.3**	**23**	**mg/kg bw/d**	**Yes**		Rat, 2gen TOX2003-1847 (Anonymous 1992c)
**(4)**	**Androstenedione ↑**	–	**90**	**mg/kg bw/d**	**Yes**	Starting at lowest dose, however: low dose selected as effect dose; lower dose (90 mg/kg bw) not sig	Rat, hormone conc. study, TOX2003-1857 (Anonymous 1992a)
**(5)**	**Estradiol ↓**	–	**90**	**mg/kg bw/d**	**Yes**	Starting at lowest dose, however: low dose selected as effect dose; lower dose (90 mg/kg bw) not sig	Rat, hormone conc. study, TOX2003-1857 (Anonymous 1992a)
**(6)**	**Testosterone ↑**	–	**90**	**mg/kg bw/d**	**0**	Starting at lowest dose, however: low dose selected as effect dose; only lower dose (90 mg/kg bw) sig	Rat, hormone conc. study, TOX2003-1857 (Anonymous 1992a)
**(7)**	**FSH ↑**	–	**90**	**mg/kg bw/d**	**Yes**	Starting at lowest dose, however: low dose selected as effect dose	Rat, hormone conc. study, TOX2003-1857 (Anonymous 1992a)
**(8)**	**Testosterone level ↑**	**15**	**50**	**mg/kg bw/d**	**Yes**	No effect on fetal toxicity levels in male and female analyzed in the same study; no effect on other steroids	In vivo mechanistic study (Taxvig et al. 2007)

*bw* body weight; *carc*. carcinogenicity; *conc*. concentration; *FSH* follicle stimulating hormone; sig. = significant; *LOAEL* lowest observed (adverse) effect level; *NOAEL* no observed (adverse) effect level; ↑ = increase; ↓ = decrease

Both propiconazole and epoxiconazole have clear effects on steroidogenesis with aromatase (CYP19a1) being one of the primary molecular targets. Yet, other enzymes relevant for steroidogenic metabolism are affected too—one being steroid-17alpha-hydroxylase (CYP17a1). The underlying multi-enzyme inhibition has been confirmed in vivo as well as in vitro and explains the apparent variety of effects observed for azoles such as altered levels of testosterone and/or estradiol or changes in progesterone, prolactin, aldosterone, ACTH, cortisol and/or androstenedione (Draskau and Svingen 2022).

Furthermore, some triazoles have been reported to be potent AR inhibitors (Kjærstad et al. 2010) and so were epoxiconazole and propiconazole with the first being more effective than the latter.

The overlapping actions of this class of fungicides make it difficult to attribute observed adverse endocrine effects to a specific MoA, although a link to a general underlying disruption of sex-hormone metabolism is plausible (Draskau and Svingen 2022; Marx-Stoelting et al. 2014; Taxvig et al. 2013).

### Discussion of threshold for adversity relevant for the ED identification

The studies evaluated for the ED assessment of epoxiconazole as well as propiconazole were mainly GLP-compliant studies performed in accordance with specific OECD Test Guidelines (see Tables 7, 8, 9, 10). Similar to dimethomorph, for each study (with one exception) with reported endocrine adversity, three doses were tested along with a proper control group, and the interval between doses was appropriately spaced between 3- and tenfold. Therefore, the studies were considered sufficiently reliable for a threshold assessment regarding respective endocrine adversity (Tables 7, 8, 9, 10). For propiconazole, the most sensitive ED-related effects observed in vivo were estrous cycle disturbances and a decrease in absolute testis weights (LOAEL: appr. 192 mg/kg bw/d, NOAEL: appr. 42 mg/kg bw/d) in a two-generation reproduction study in rats (Anonymous 1985). The most sensitive relevant effects observed in epoxiconazole studies were vaginal hemorrhages, a decreased gestation index and a prolonged pregnancy (LOAEL 23 mg/kg bw/d (Anonymous 1992f)) and ovarian cysts (LOAEL 8 mg/kg bw/d (Anonymous 1992g) in females as well as a reduced fertility index and a prolonged cohabitation time in males at a LOAEL of 23 mg/kg bw/d (Anonymous 1992f). The NOAEL for these effects in both studies (Anonymous 1992f, 1992g) was 2.3 and 2 mg/kg bw/d, respectively.

Besides the OECD-compliant studies there are results from a number of mechanistic assays available (Goetz et al. 2009; Laville et al. 2006; Ohno et al. 2004; Sanderson et al. 2002; Taxvig et al. 2013; Vinggaard et al. 2000). In several cases these assays were conducted in vitro with five or more dose levels tested. As observed with the in vivo data these tests generally demonstrate a clear dose–response relationship, indicating a no effect level (see Tables 7, 8, 9, 10). The exception are those few cases where the respective lowest dose measured was still in the effect range (*e.g.*, 1 µM in aromatase assays; Table 7).

As for dimethomorph the overall evidence strongly supports a threshold for the endocrine effects of propiconazole and epoxiconazole. However, it should be noted that for substances with such a plethora of overlapping MoAs any threshold will inherently be an effect threshold free of any further mechanistic implications.

#### Metiram—thyroid-disruption

Metiram (CAS 9006–42-2; zinc ammoniate ethylenebis(dithiocarbamate)-poly[ethylenebis(thiuramdisulphide)] belongs to the dithiocarbamates and is a non-systemic fungicide. It was first discovered in the 1960s by BASF and is registered in Europe since the late 80ies. Its predominant use is in PPPs for foliar spray in grapes and potatoes against *Plasmopara viticola, Guignardia bidwellii, Pseudopezicula tracheiphila*, *Phytophthora infestans* and *Alternaria* spp (Italy 2019). As active substance metiram is currently under renewal evaluation, providing it with an extensive up-to-date in vivo data set consisting of several studies from multiple species (rat and dog). The ongoing assessment identified ethylenethiourea (ETU), a metabolite and degradation product of metiram, together with several other metabolites as likely to cause T-mediated adverse effects. Given the updated dataset and modality metiram hence was selected as a third for this study.

The RAR for metiram was prepared in accordance with Regulation (EU) No 2018/1659 (EU 2018a) and put forward for peer review by EFSA’s Pesticide Peer Review Experts´ Meeting (EFSA 2023b). The dataset available for metiram mainly consists of the standard OECD test guideline studies. In particular, metiram was assessed in two short-term (90-day) and a 2-generation reproduction toxicity studies in rats, a 19-week and a 1-year dog study, and a 90-day mouse study. With regard to the relevant metabolite ETU the dataset includes studies with ETU only as well as spiking studies with metiram with 0.2, 2 or 2.2% of ETU added, respectively. ETU is a metabolite, contaminant, and an impurity of metiram having a direct effect on the synthesis of thyroid hormones by thyroid peroxidase (TPO) inhibition (Doerge and Takazawa 1990; Freyberger and Ahr 2006 *apud (*Italy 2019*)*. Data from bile cannulation (Wenker and Krebbers 2015 *apud (*Italy 2019*)*) indicate approximately 7.5% of orally administered to be converted to ETU. Therefore, although generally not quantified further exposure to ETU can be anticipated in all in vivo studies with metiram (Italy 2019).

Other metabolites identified during metiram metabolism studies are ethylenebisisothiocyanatesulfide (EBIS), ethyleneurea (EU), ethylenediamine (EDA), N-(2-aminoethyl)acetamide (n-acetyl EDA), 1-(4,5-dihydro-1H-imidazol-2-yl)-2-imidazolidinethione (Jaffe’s Base) and 2,3,7,8-tetrahydrodiimidazo[2,1-b:1’,2’-e][1,3,5]thiadiazine-5-thione (TDIT) (EFSA 2020; Italy 2019). The underlying pathway sees metiram converted to ETU presumably directly or indirectly via TDIT and EBIS. ETU is then transformed into EU with the generation of other minor metabolites via EBIS methylation being postulated. While there is no toxicological information available for all metabolites repeated dose studies with EBIS as well as with EU show that they can also induce thyroid hypertrophy and hyperplasia (EFSA 2020; Italy 2019).

Substance-induced histopathological changes in the thyroid and changes in thyroid hormones (THs) and thyroid-stimulating hormone (TSH) were observed in three species (rat, mouse and dog) at doses of metiram that were considered to be at or below the maximum tolerated dose (MTD) (Table 11). In rat dose–response relationships were (partially) observed with regard to hormone measurements in a 2-generation study. It should be noted in this context that while the corresponding study failed to record a significant effect on PND 21 the magnitude of the effect was unambiguously higher on PND 21 than on PND 4. Given the statistical significance of the latter it therefore seems questionable to discard the observations made on PND 21. The more so as the same study saw thyroid follicular cell hypertrophy/hyperplasia incidences dose dependently increased in F_0_ males and F_1_ adult males and females, respectively. Further in vivo data indicate an effect of ETU on brain morphometry (EFSA 2023b). In contrast to the extensive data in vivo mechanistic studies in vitro are very limited, most likely due to the rapid transformation and degradation of metiram in such systems (EPA 2005).

**Table 11:** Thyroid effects identified at metiram studies - effects considered relevant for ED identification are marked in bold

No	Effect	NOAEL	LOAEL	Unit(s)	Dose–response	Remarks	Study
**(1)**	**Thyroid follicular cell hypertrophy/hyperplasia** **(F0 male and F1 adult male and female)**	**–**	**9**	**mg/kg bw/day**	**Yes**		2-generation rat (Anonymous 2011)
(2)	Thyroid weight ↑ (absolute and relative, F0 and F1 adults, male and female)	31	92	mg/kg bw/day	No		2-generation rat (Anonymous 2011)
**(3)**	**T4 ↓ (F0 male and female)**	**9**	**31**	**mg/kg bw/day**	Partially*	*T4 ↓ in mid and high doses, but not proportionally in both sexes	2-generation rat (Anonymous 2011)
**(4)**	**T4 ↓ (F1 pup female PND4)**	**31**	**92**	**mg/kg bw/day**	**No**		2-generation rat (Anonymous 2011)
**(5)**	**T3 ↓ (F1 pup males PND21)**	**–**	**9**	**mg/kg bw/day**	**Yes**		2-generation rat (Anonymous 2011)
**(6)**	**TSH ↑ (F0 and F1 males)**	**9**	**31**	**mg/kg bw/day**	**No in F0 and yes in F1 males, respectively**		2-generation rat (Anonymous 2011)
**(7)**	**TSH ↑ (F1 pup female PND4)**	**31**	**92**	**mg/kg bw/day**	**No**		2-generation rat (Anonymous 2011)
**(8)**	**Thyroid follicular cell hyperplasia, male and female**	**2.59**	**29.9**	**mg/kg bw/day**	**Yes**	Dose-related in severity, but no table is available, only description 2.59 mg/kg bw/day = 80 ppm; 29.9 mg/kg bw/day = 1000 ppm; 84.8 mg/kg bw/day = 30,000 ppm Using BMDL20 a value of 5.2 mg/kg bw (162 ppm) is derived	1-year dog study (Anonymous 1991a)
**(9)**	**T4↓ male and female**	**2.59**	**29.9**	**mg/kg bw/day**	**Yes**	In males dose–response on 3 points of collection time (13, 26 and 52 weeks). In females, not always statistically sig at high dose 3000 ppm in different time points, but reduced at the LOAEL too (1000 ppm)	1-year dog study (Anonymous 1991a)
(10)	Thyroid weight ↑ (absolute and relative, bilateral) male and female	29.9	84.8	mg/kg bw/day	Yes#	Slight to marked ↑ in group mean thyroid gland weight in males and females at 1000 ppm or 3000 ppm*, respectively #Not statistically sig at 1000 ppm, but ↑ already	1-year dog study (Anonymous 1991a)
**(11)**	**Thyroid follicular cell hyperplasia, female only**	**17 (F)**	**71 (F)**	**mg/kg bw/day**	**No#**	#Moderate severity, but no incidence table available 13 mg/kg bw/day M, 17 mg/kg bw/day F= 200 ppm; 59 mg/kg bw/day M, 71 mg/kg bw/day ***F*** = 900 ppm	90-day neurotoxicity rat study (Anonymous 2014a)
**(12)**	**T4↓** **male and females**	**13 (M)** **17 (F)**	**59 (M),** **71 (F)**	**mg/kg bw/day**	**No**	For females, reduction was also identified at the mid dose, but not statistically sig	90-day neurotoxicity rat study (Anonymous 2014a)
(13)	Thyroid weight ↑ (absolute and relative), female only	17 mg/kg bw/day	71 mg/kg bw/day F	mg/kg bw/day	No	Only relative weight was statistically sig in females	90-day neurotoxicity rat study (Anonymous 2014a)
(14)	Thyroid toxicity (not further described) male and female	16	55	mg/kg bw/day	Not possible to evaluate, no tabulated results	16 mg/kg bw/day = 400 ppm; 55 mg/kg bw/day = 1600 ppm	19-week dog (Anonymous 1989a)
**(15)**	**Thyroid hypertrophy and vacuolization** **male and female**	**400**	**1200**	**mg/kg bw/day**	**Yes**	No tabulated table available, only description text 100 mg/kg bw/day = 300 ppm; 400 mg/kg bw/day = 1000 ppm; 1200 mg/kg bw/day = 3000 ppm; 3000 mg/kg bw/day = 7500 ppm	3-month mouse (Anonymous 1992b)
**(16)**	**T4↓** **male and female**	**100**	**400**	**mg/kg bw/day**	**Yes**	No tabulated table available, only description text	3-month mouse (Anonymous 1992b)
(17)	T3↑ male	1200	3000	mg/kg bw/day	No	No tabulated table available, only description text	3-month mouse (Anonymous 1992b)
**(18)**	**Thyroid hypertrophy/hyperplasia,** **male**	**–**	**41**	**mg/kg bw/day**	**Yes**	No tabulated values available, only description text, but only 2 doses tested, 41 and 126 mg/kg bw	28-day comparative toxicity study in rats (Anonymous 2014b)
(19)	Thyroid weight ↑, relative male	41	126	mg/kg bw/day	No	No tabulated values available, only description text	28-day comparative toxicity study in rats (Anonymous 2014b)
**(20)**	**T4↓** **male**	**–**	**41**	**mg/kg bw/day**	**Yes**	No tabulated values available, only description text	28-day comparative toxicity study in rats (Anonymous 2014b)
(21)	T3↑ male	41	126	mg/kg bw/day	No	T3 levels ↑ of marginal significance (< 15%). No tabulated values	28-day comparative toxicity study in rats (Anonymous 2014b)
(22)	Thyroid weight ↑, relative male and female	23.5 (M), 27.3 (F)	73.9 (M), 88.8 (F)	mg/kg bw/day	No	Absolute weight ↑ also in males at 960 ppm, but not statistically sig 0.4 mg/kg bw/day (M, F) = 5 ppm 23.5 mg/kg bw/day M, 27.3 mg/kg bw/day *F* = 320 ppm 73.9 mg/kg bw/day M, 88.8 mg/kg bw/day *F* = 960 ppm	90-day rat study (Anonymous 1992e, 2015)
**(23)**	**T4↓** **male and female**	**23.5 (M), 27.3 (F)**	**73.9 (M), 88.8 (F)**	**mg/kg bw/day**	**No**	In F ↓ at 320 ppm, but not statistically sig	90-day rat study (Anonymous 1992e, 2015)
**(24)**	**TSH↑** **male and female**	**–**	**0.4**	**mg/kg bw/day**	**Yes#**	#Not always dose–response, but in every dose. More evident in females than males	90-day rat study (Anonymous 1992e, 2015)
**(25)**	**T3↑** **male**	**5.8**	**23.5**	**mg/kg bw/day**	**Yes#**	#↑ at 2 highest doses, but not proportionally/higher at high dose 5.8 mg/kg bw/day = 80 ppm; 3.5 mg/kg bw/day = 320 ppm	90-day rat study (Anonymous 1992e, 2015)

*bw* body weight; *carc*. carcinogenicity; *conc*. concentration; *F* female; *LOAEL* lowest observed (adverse) effect level; *M* male; *NOAEL* no observed (adverse) effect level; *sig* significant; *TSH* thyroid-stimulating hormone; ↑ = increase; ↓ = decrease

Even so the overall data are sufficiently conclusive as to indicate the adverse effects of metiram to be T-mediated (EFSA 2023b). There is empirical support that exposure to metiram causes dose-dependent inhibition of TPO due to the formation of ETU. The resulting inhibition of thyroid hormone synthesis then leads to hypothyroidism, *i.e.,* an increased pituitary TSH release, eventually manifesting in follicular hypertrophy and hyperplasia both of which are known neoplastic precursors in the thyroid.

### Discussion of threshold for adversity relevant for the ED identification

Despite its limitations regarding an assessment according to the ECHA/EFSA ED guidance (ECHA/EFSA 2018) the data set on metiram is sufficiently comprehensive to conclude on the existence of endocrine effect thresholds. However, at least with regard to metiram the underlying toxicology does not allow conclusions on single substances as any thyroid effects observed could be either related to metiram and/or its metabolite ETU (EFSA 2023b).

For ETU the lowest recorded NOAEL for thyroid histological endpoints is 0.2 mg/kg bw/d based on a 1-year dog study (thyroid histopathology). This is supported in rat by an extended one-generation study (thyroid histopathology and hormones) and a two-generation study with a parental NOAEL of 0.2 mg/kg bw day 0.27 mg/kg bw day, respectively. However, another study reported thyroid hyperplasia in rat at 0.25 mg/kg bw day after 24 months, indicating some remaining uncertainties for the use of 0.2 mg/kg bw day as a NOAEL (EFSA 2023b).

Contrastingly the lowest recorded NOAEL for metiram is 2.6 mg/kg bw. Again, this is based on the observed thyroid effects (thyroid hormones, thyroid weight and histopathological changes) from the 1-year dog study (EFSA 2023b). In this study, the substance-induced decrease of T4 in males was dose-dependent (13, 26 and 52 weeks). In females the respective measurements for T4 were not always statistically significant at the high dose of 84.8 mg/kg bw for all different time point but were nevertheless clearly reduced at the LOAEL (29.9 mg/kg bw). In rat the lowest LOAEL where thyroid histopathology (thyroid follicular cell hypertrophy and hyperplasia in parental animals, *i.e.,* F_0_ males and F_1_ adult males and females) was observed is 9 mg/kg bw day in adult animals, based on a two-generation study. Considering hypothyroidism as the adverse outcome (AO) for metiram, a threshold for each individual Key Event (KE) in vivo can be derived from the 1-year dog metiram study (T4 changes, relative thyroid weight changes and increase severity of thyroid follicular cell hyperplasia) at male and female animals. As for ETU, it causes similar effects on the thyroid hormonal system in rats, mice and dogs, although quantitative differences are seen in dose–response. However, ETU T4 effects in rats are in the same range as for the lowest NOAEL for metiram in dogs. Overall, therefore there is strong evidence that metiram thyroid toxicity (hypothyroidism) follows a dose-dependent threshold behavior.

## Discussion

Endocrine disruption represents a particular complex endpoint. That is not the least due to the fact that it actually is not an endpoint in the classical sense but a mechanistic concept. This poses particular challenges as the involvement of multiple and quite diverse mechanisms not only requires an in-depth analysis of the available data, but also a profound mechanistic understanding of the underlying molecular biology and biochemistry. This introduces additional uncertainties into the respective assessments which is inherently at odds with the regulatory ideal (and quest) of standardized decision finding. Considering that for substances with potentially disrupting effects on the hormone system there are usually high health issues at stake (*i.e.*, carcinogenicity, reproductive toxicity, developmental toxicity) a high degree of conservatism when assessing such substances not only seems appropriate but should be warranted. This, together with a frequent lack of data and methodological challenges has historically contributed to a regulatory “no threshold”-approach for substances with ED properties.

However, originally rooted in pragmatism today’s discussion sees this increasingly mistaken as factual often adding the specter of uncertainties (some real, some less real) for justification. This is in so far problematic as that this is not only unscientific and because potential “EDs” routinely constitute great parts of our environment and diets (and always have done so, not the least in form of natural ingredients) but for the fact that EDs, as all other substances, are subject to increasingly sensitive analytical detection. At the current pace of analytical development, we will soon come to the point where we will be able to measure “everything everywhere”. Apart from the paradoxical situation that we will then have to continuously reassess even the health wise most unsuspicious foods and commodities it poses the vital question if under these circumstances a universal “no threshold” approach really still is the way to go, given that often new methods and data are available. The same holds true for substances where there is either no alternative or where endocrine disruption is part of the function—how can we assure their safe and functional regulation?

As outlined earlier in this paper as well as by others such as Autrup et al. (2016) there is no reason to believe that endocrine mechanisms are subject to a different biochemistry or exception from thermodynamics. Based on the latter every substance induced effect should have an effectual threshold, at least theoretically. Practically this threshold can of course be outside of practical or experimental accessibility. Alternatively, it also might be that for certain cases and scenarios a threshold-based assessment and regulation simply does not appear appropriate or feasible.

To draw a conclusion on the existence of ED properties of a substance the dose–response relationship for both endocrine activity and any resulting adversity at individual or population level as well as the dose dependency of the mechanistic link between these elements has to be analyzed in order to support a quantitative assessment. Conducting an ED assessment with high confidence requires critical review of data-rich substances including a full set of experimental studies. Because of the extensive toxicity data requirements (*e.g.*, with regard to assessing reproductive toxicity and chronic toxicity/carcinogenicity) active substances reviewed under the PPPR and BPR for approval in the EU can be considered as ideal subjects for further exploring the existence of dose–response relationships of endocrine-mediated effects.

The ED assessments of 12 active substances for plant protection or biocidal use completed under the remit of EFSA and ECHA, respectively, were screened with regard to the quality and adequacy of data for determining a threshold for endocrine effects. In the screening exercise, technical and conceptual challenges were identified with nine substances which in consequence were deemed non-suitable for an in-depth case study. This could be because the uncertainties identified in the ED assessments were too high, e.g., in the establishment of the biological plausibility between endocrine activity and adversity or due to insufficient investigation of endocrine activities in the overall data package. For example, with asulam, no consistent pattern of thyroid toxicity was observed in the in vivo studies and the evidence between the molecular initiating event (MIE) and the AO was considered weak as the KEs were equivocal. Meanwhile benthiavalicarb showed multiple MoAs which potentially contribute mutually to the observed carcinogenicity making it difficult to establish a clear dose–response and AOP. Lastly, for clofentezine, there still is high uncertainty whether the observed thyroid effects in male rats are secondary to liver toxicity or relevant to human health (due to species-specific metabolism). However, in absence of further mitigating mechanistic data clofentezine has been identified as an EDC.

In the end, four endocrine disrupting chemicals were deemed suitable and sufficiently backed up by data as to go forward as case studies to investigate respective dose–response relationships for ED-related parameters and to draw a conclusion about the existence of potential thresholds. Namely these were dimethomorph, propiconazole and epoxiconazole (forthwith grouped as “azoles”), and metiram. Epoxiconazole was not initially included in the screening exercise as no formal ED assessment was performed with this substance but was added alongside propiconazole to strengthen the case on azoles. These case studies feature active substances with distinctive endocrine modes of actions (*e.g.*, androgen or thyroid). They inherently still have some degree of uncertainties and/or data gaps in their ED assessments, such as involvement of multiple endocrine modes of action or limited data on the investigation of endocrine activity. However, what distinguishes these case studies from the other non-suitable substances is the consistent pattern of endocrine adversities observed across multiple studies. Within this pattern threshold levels could be identified for all four substances, either directly for the endocrine adversity or as effectual threshold.

This critically extends and confirms the results of previous work (Marx-Stoelting et al. 2014), where summaries for approximately 300 regulatory toxicity studies were evaluated. The results of this previous exercise revealed that but for a few studies NOAELs could be established. In follow-ups with lower doses, it was subsequently shown that it was usually possible to derive NOAELs for endocrine-related effects and to confirm effects and respective NOAELs in other studies within the extensive data packages available for active substances under PPPR (Marx-Stoelting et al. 2014). Altogether the question therefore is not if there is a threshold for endocrine disrupting effects but if there are sufficient data or suitable experimental means for assessing the corresponding effects.

There is an acknowledged shortfall of validated test methods for comprehensively testing for potential endocrine adversity (Fritsche et al. 2023; OECD 2018; Solecki et al. 2017). While this has been subject to repeated discussions, not to say at times bemoaning, this should not make forget that a) for a substantial number of substances the available data already may well allow substantial assessments, also for endocrine effects, b) that these assessments can profit from often extensive sets of complementary data in vitro and c) that there is a lot of work in progress for improving the situation. This includes a large set of in vitro methods for thyroid hormone disruption (THD) being in the pipeline to become validated, based on the OECD scoping paper (OECD 2014) and with input from THD meetings and workshops (EU-NETVAL 2023). Also, AOP-informed integrated approach to testing and assessment (IATA) case studies to answer a developmental neurotoxicity (DNT) hazard identification and characterization have been exercised to support the regulatory decisions for pesticide active substances (EFSA 2021a). Other initiatives for improving the situation include major projects in the US (*i.e.,* Tox21 ((Krewski et al. 2010); see also https://tox21.gov), ToxCast ((Reif et al. 2010); see also https://www.epa.gov/comptox-tools/toxicity-forecasting) and EDSP ((Browne et al. 2017) see also https://www.epa.gov/endocrine-disruption/endocrine-disruptor-screening-program-edsp-21st-century)), and the EU such as PARC ((Marx-Stoelting et al. 2023); https://www.eu-parc.eu/), EU-ToxRisk ((Moné et al. 2020); https://eu-toxrisk.eu/), RiskHunt3R ((Pallocca et al. 2022); https://www.risk-hunt3r.eu/) as well as other projects of the ASPIS cluster (https://aspis-cluster.eu/) which put their efforts into an improved regulatory use of NAMs.

It is particularly the latter which will help to also address issues such as critical windows of exposure and the repeatedly brought up question if and how mixtures (intended and unintended) might contribute to endocrine effects (or not) (*e.g.*, (Demeneix et al. 2020). One can only but speculate. However, while the issue of temporality (exposure to EDCs during critical windows of development) is biologically well supported to be a potential cause of concern, this is not necessarily the case for mixtures, at least not in a generalized way. The topic of mixtures has intensively and been reviewed and discussed elsewhere (Kortenkamp 2007; Tralau et al. 2021). With regard to concerns about the uncertainty of the assessment and evaluation of chemical mixtures, particularly for EDs, we would like to raise two points if and how potential endocrine disruptors should be considered and/or addressed. That is a), that for any substance-triggered effect to occur respective (co-)exposure has to occur either simultaneously or in sufficiently close temporal succession and b), for the corresponding effects to become regulatorily relevant the respective concentrations would need to be high enough as to exceed the usual safety factors and would need to do so in presence of organismic detoxification. Realistically one would expect this to be the case only for few, mostly synergistically acting, cases. Particularly so, as exposure to (natural occurring) toxicologically active substances is a biologically common phenomenon (Hodges and Minich 2015).

Therefore, while the assessment of any critical effect, including those caused by EDs should always rely on strong evidence and mechanistic understanding it would appear wrong to conclude assessments based on existing regulatory studies to be necessarily insufficiently protective for ED.

Indeed, the present study demonstrates otherwise. Also, while additional mechanistic data always are preferable and helpful for a profound assessment, they are not necessarily mandatory as shown for metiram. Altogether the key to endocrine assessments rather is data that are reliable and rational. This is also substantiated by the fact that it was mostly lack of data which for a majority of the screened substances in this analysis prevented carry on of the respective compounds as case subjects.

Besides the studies presented here there are other examples of ED-threshold assessments, such as the assessment for atrazine by the US EPA, which was also used as model substance to analyze complex interrelationships between the immune, endocrine, and nervous systems (Galbiati et al. 2021). Vandenberg et al. (2020) reviewed three model agrochemicals for estrogen signaling pathways. Namely these were DDT, endosulfan as well as atrazine all of which can take effect on a molecular level in distinct ways to ultimately produce estrogenic responses. Moreover a plethora of pharmaceuticals is designed to specifically alter estrogenic pathways in a dose-responsive manner, e.g., for hormone replacement therapy, birth control, or treatment of estrogen-dependent cancers. Borgert et al. (2018) presented an empirical method for determining a human-relevant potency threshold for ERα agonistic substances such as genistein or tamoxifen to “*distinguish between chemicals that may be capable of, versus those likely to be incapable of, producing adverse effects in humans *via* the specified MoA*”.

In addition to testing-related issues there also is a need for agreement and guidance on the general approach for setting a suitable point of departure for endocrine disruption. This is illustrated by the case of the biocidal active substance cyanamide. Subsequent to the identification of this substance as an ED, there were several questions referred by the European Commission to ECHA and the evaluating competent authority, all of which related to a quantitation of the potential risk in form of a threshold (ECHA 2021b). Yet, and despite an extensive dataset and in-depth mechanistic understanding of the underlying thyroid MoA, the responsible working group decided, that a quantitative risk assessment should not be supported due to a missing study on the downstream effect of developmental neurotoxicity, lack of data on other unrelated modes of action and, in particular, lack of a guidance on ED risk assessment (ECHA 2021b, 2021c). Formally correct this focus on hazard completely omitted the toxicologically equally important question though under which conditions cyanamide would indeed pose a risk for human health and the environment. To overcome this, it is necessary to stop at the mere identification of hazard (as foreseen in the current ECHA/EFSA ED guidance (ECHA/EFSA 2018)) but to amend the process of ED-assessment as to also cover hazard characterization. Considering the prominent role of the plausible link between endocrine activity and adversity in the definition of EDs, we suggest that such a guidance should assume a mechanism/AOP-based perspective and explore the potential of quantitative AOPs. For example, the definition of a threshold for any of the upstream key events in a well understood mode of action could be sufficient to support a threshold for any downstream event including the adverse outcome at corresponding or higher doses (concentrations). In the case of cyanamide (ECHA 2021c), the demonstration of a level that does not affect thyroid hormone synthesis and levels (to a detectable degree) would then be sufficient to postulate a safe level also with regard to developmental neurotoxicity, making the conduct of the requested in vivo DNT studies and the associated use of animals unnecessary.

## Summary and conclusion

Altogether the presented case studies demonstrate that threshold levels (*e.g.*, NOAELs) can well be identified also for substances with endocrine adversity, if there is reliable data and high-quality studies available. Moreover, this paper provides an in-depth understanding on the type of information required for the clear identification of a threshold/NOAEL for ED adversity.

In principle, the concepts and issues identified in the context of this study should likewise apply to the assessment of other potential endocrine disruptive chemicals. Following a tiered approach can and would decrease the necessity of further animal studies in this context to a minimum. Without the need for further development there is already the possibility of using existing data for such assessments. In addition, there is a range of initiatives for implementing more endocrine endpoints into standard regulatory animal studies together with the option of increased regulatory use and further development of reliable in silico and in vitro methods as to gain necessary mechanistic information.

In light of the challenges of responsible and safe substance use, increasing analytical sensitivity and regulatory consistency it seems scientifically questionable not if but rather for how long any “no threshold”-concepts will still be sustainable, pragmatic and convenient as they might still seem. Backed up by the methodological and scientific progress of the past years we should therefore think now about how risk assessment could - and should - be implemented also for endocrine disruptors.

## Data Availability

Data analysed in this study was compiled based on the data available for the respective substances. All analyses and essential data are included in this published article.
